# 2-(2-Pyrrolidinio)-1*H*-benzimidazol-3-ium dinitrate

**DOI:** 10.1107/S1600536809018558

**Published:** 2009-05-23

**Authors:** Jing Dai

**Affiliations:** a Ordered Matter Science Research Center, College of Chemistry and Chemical Engineering, Southeast University, Nanjing 210096, People’s Republic of China

## Abstract

In the title compound, C_11_H_15_N_3_^2+^·2NO_3_^−^, one of the imidazole N atoms and the N atom of the pyrrolidine ring are protonated. The pyrrolidine ring adopts an envelope conformation, with the C atom carrying the benzoimidazolium substituent as the flap atom. In the crystal structure, cations and anions are linked through N—H⋯O hydrogen bonds, forming chains that run parallel to the *c* axis.

## Related literature

For background to the applications of proline derivatives, see: Fu *et al.* (2007 ▶); Aminabhavi *et al.* (1986 ▶). For the structures of metal complexes with ligands similar to the title compound, see: Dai & Fu (2008*a* ▶,*b* ▶); Fu & Ye (2007 ▶).
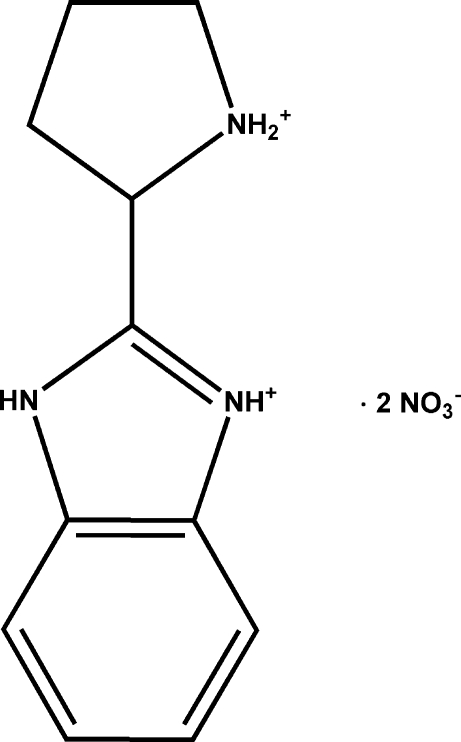


## Experimental

### 

#### Crystal data

C_11_H_15_N_3_^2+^·2NO_3_^−^
*M* *_r_* = 313.28
Monoclinic, 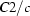

*a* = 22.078 (2) Å
*b* = 11.154 (1) Å
*c* = 14.670 (1) Å
β = 127.18 (1)°
*V* = 2878.3 (4) Å^3^
*Z* = 8
Mo *K*α radiation
μ = 0.12 mm^−1^
*T* = 298 K
0.35 × 0.30 × 0.15 mm

#### Data collection

Rigaku Mercury2 diffractometer
Absorption correction: multi-scan (*CrystalClear*; Rigaku, 2005 ▶) *T*_min_ = 0.959, *T*_max_ = 0.982
14543 measured reflections
3276 independent reflections
2195 reflections with *I* > 2σ(*I*)
*R*_int_ = 0.048

#### Refinement

*R*[*F* ^2^ > 2σ(*F* ^2^)] = 0.059
*wR*(*F* ^2^) = 0.144
*S* = 1.09
3276 reflections
199 parameters
H-atom parameters constrained
Δρ_max_ = 0.31 e Å^−3^
Δρ_min_ = −0.19 e Å^−3^

### 

Data collection: *CrystalClear* (Rigaku, 2005 ▶); cell refinement: *CrystalClear*; data reduction: *CrystalClear*; program(s) used to solve structure: *SHELXS97* (Sheldrick, 2008 ▶); program(s) used to refine structure: *SHELXL97* (Sheldrick, 2008 ▶); molecular graphics: *SHELXTL* (Sheldrick, 2008 ▶); software used to prepare material for publication: *SHELXTL*.

## Supplementary Material

Crystal structure: contains datablocks I, global. DOI: 10.1107/S1600536809018558/sj2610sup1.cif

Structure factors: contains datablocks I. DOI: 10.1107/S1600536809018558/sj2610Isup2.hkl

Additional supplementary materials:  crystallographic information; 3D view; checkCIF report

## Figures and Tables

**Table 1:** Hydrogen-bond geometry (Å, °)

*D*—H⋯*A*	*D*—H	H⋯*A*	*D*⋯*A*	*D*—H⋯*A*
N3—H3*A*⋯O4^i^	0.86	1.93	2.788 (2)	177
N3—H3*A*⋯O5^i^	0.86	2.50	3.020 (2)	120
N5—H5*B*⋯O1^i^	0.90	1.89	2.771 (2)	167
N5—H5*B*⋯O3^i^	0.90	2.64	3.149 (2)	117
N5—H5*A*⋯O5^ii^	0.90	1.90	2.768 (2)	162
N4—H4*A*⋯O1	0.86	2.04	2.850 (2)	157
N4—H4*A*⋯O2	0.86	2.42	3.121 (3)	139

Symmetry codes: (i) 
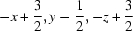
; (ii) 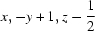.

**Fig. 1.**
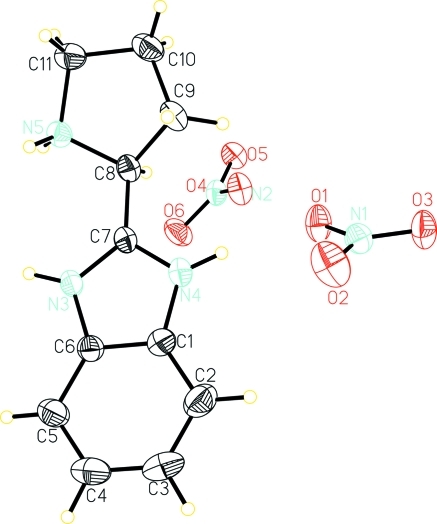
A view of the title compound with the atomic numbering scheme. Displacement ellipsoids are drawn at the 30% probability level.

**Fig. 2.**
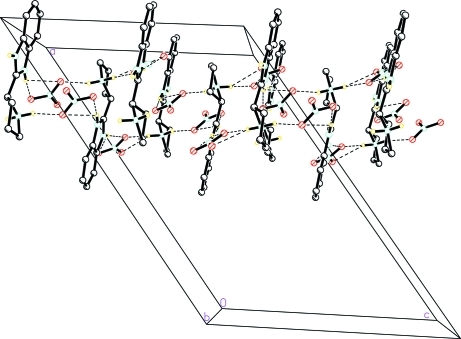
Partial packing view of the title compound showing the formation of a chain parallel to the c axis. All H atoms not involved in hydrogen bonding (dashed lines) were omitted for clarity.

## supplementary crystallographic information

### Comment

Heterocyclic amine derivatives have found wide range of applications in material science and display ferroelectric, fluorescence and dielectric behaviors. There has also been an increased interest in the preparation of coordination compounds from these heterocyclic ligands (Aminabhavi *et al.*, 1986; Dai & Fu, 2008*a*,*b*; Fu & Ye, 2007; Fu *et al.*, 2007). We report here the crystal structure of the title compound, (I), 2-(pyrrolidinium-2-yl)-1*H*-benzo[*d*] imidazol-3-ium dinitrate.

In the title compound, (C_11_H_15_N_3_)^2+^.2(NO_3_)^-^, the N4 atom of the imidazole and the N5 atom of pyrrolidine ring are protonated. The pyrrolidine ring adopts an envelope conformation with the C8 atom carrying the benzoimidazolium substituent as the flap atom. In the crystal structure, cations and anions are linked through N—H···O hydrogen bonds forming chains that run parallel to the *c* axis. (Fig. 2, Table 1).

### Experimental

The homochiral ligand *S*-2-(pyrrolidin-2-yl)-1*H*-benzo[*d*]imidazole was synthesized by reaction of *S*-pyrrolidine-2-carboxylic acid and benzene-1,2-diamine according to the procedure described in the literature (Aminabhavi *et al.*, 1986). *S*-2-(pyrrolidin-2-yl)-1*H*-benzo[*d*]imidazole (3 mmol) was dissolved in distilled water (20 ml) and nitric acid (1 ml). The solution was evaporated in air affording colorless block-like crystals of (I) suitable for X-ray analysis.

### Refinement

All H atoms attached to C and N atoms were fixed geometrically and treated as riding with C—H = 0.93 Å (aromatic), 0.97 Å (methylene) or 0.98 Å (methine) and N—H = 0.90 Å (N5), 0.86 Å (N3, N4) with *U*_iso_(H) = 1.2*U*_eq_(C,N).

### Crystal data

**Table:**

C_11_H_15_N_3_^2+^·2NO_3_^−^	*F*(000) = 1312
*M**_r_* = 313.28	*D*_x_ = 1.446 Mg m^−^^3^
Monoclinic, *C*2/*c*	Mo *K*α radiation, λ = 0.71073 Å
Hall symbol: -C 2yc	Cell parameters from 3275 reflections
*a* = 22.078 (2) Å	θ = 3.2–27.5°
*b* = 11.154 (1) Å	µ = 0.12 mm^−^^1^
*c* = 14.670 (1) Å	*T* = 298 K
β = 127.18 (1)°	Block, colourless
*V* = 2878.3 (4) Å^3^	0.35 × 0.30 × 0.15 mm
*Z* = 8	

### Data collection

**Table:**

Rigaku Mercury2 diffractometer	3276 independent reflections
Radiation source: fine-focus sealed tube	2195 reflections with *I* > 2σ(*I*)
graphite	*R*_int_ = 0.048
Detector resolution: 13.6612 pixels mm^-1^	θ_max_ = 27.5°, θ_min_ = 3.2°
ω scans	*h* = −28→28
Absorption correction: multi-scan (*CrystalClear*; Rigaku, 2005)	*k* = −14→14
*T*_min_ = 0.959, *T*_max_ = 0.982	*l* = −18→19
14543 measured reflections	

### Refinement

**Table:**

Refinement on *F*^2^	Primary atom site location: structure-invariant direct methods
Least-squares matrix: full	Secondary atom site location: difference Fourier map
*R*[*F*^2^ > 2σ(*F*^2^)] = 0.059	Hydrogen site location: inferred from neighbouring sites
*wR*(*F*^2^) = 0.144	H-atom parameters constrained
*S* = 1.09	*w* = 1/[σ^2^(*F*_o_^2^) + (0.0493*P*)^2^ + 2.1933*P*] where *P* = (*F*_o_^2^ + 2*F*_c_^2^)/3
3276 reflections	(Δ/σ)_max_ < 0.001
199 parameters	Δρ_max_ = 0.31 e Å^−^^3^
0 restraints	Δρ_min_ = −0.19 e Å^−^^3^

### Special details

**Table:**

Geometry. All e.s.d.'s (except the e.s.d. in the dihedral angle between two l.s. planes) are estimated using the full covariance matrix. The cell e.s.d.'s are taken into account individually in the estimation of e.s.d.'s in distances, angles and torsion angles; correlations between e.s.d.'s in cell parameters are only used when they are defined by crystal symmetry. An approximate (isotropic) treatment of cell e.s.d.'s is used for estimating e.s.d.'s involving l.s. planes.
Refinement. Refinement of *F*^2^ against ALL reflections. The weighted *R*-factor *wR* and goodness of fit *S* are based on *F*^2^, conventional *R*-factors *R* are based on *F*, with *F* set to zero for negative *F*^2^. The threshold expression of *F*^2^ > σ(*F*^2^) is used only for calculating *R*-factors(gt) *etc*. and is not relevant to the choice of reflections for refinement. *R*-factors based on *F*^2^ are statistically about twice as large as those based on *F*, and *R*- factors based on ALL data will be even larger.

### Fractional atomic coordinates and isotropic or equivalent isotropic displacement parameters (Å^2^)

**Table:**

	*x*	*y*	*z*	*U*_iso_*/*U*_eq_	
N3	0.66938 (10)	0.39290 (15)	0.56483 (15)	0.0433 (4)	
H3A	0.6966	0.3290	0.5937	0.052*	
N4	0.63804 (11)	0.58040 (17)	0.53606 (17)	0.0513 (5)	
H4A	0.6414	0.6572	0.5428	0.062*	
N5	0.83307 (9)	0.45350 (16)	0.71441 (14)	0.0429 (4)	
H5A	0.8163	0.4119	0.6506	0.052*	
H5B	0.8425	0.4016	0.7686	0.052*	
C1	0.57133 (13)	0.5163 (2)	0.4595 (2)	0.0500 (6)	
C2	0.49697 (15)	0.5521 (3)	0.3784 (3)	0.0709 (8)	
H2	0.4832	0.6325	0.3664	0.085*	
C3	0.44437 (16)	0.4628 (3)	0.3162 (3)	0.0778 (9)	
H3	0.3939	0.4834	0.2599	0.093*	
C4	0.46486 (16)	0.3430 (3)	0.3353 (2)	0.0725 (8)	
H4	0.4273	0.2856	0.2920	0.087*	
C5	0.53812 (15)	0.3057 (2)	0.4155 (2)	0.0593 (7)	
H5	0.5513	0.2250	0.4276	0.071*	
C6	0.59181 (12)	0.3961 (2)	0.47789 (19)	0.0448 (5)	
C7	0.69499 (12)	0.50468 (19)	0.59632 (18)	0.0418 (5)	
C8	0.77457 (13)	0.54335 (19)	0.6904 (2)	0.0465 (6)	
H8	0.7785	0.5530	0.7602	0.056*	
C9	0.80026 (15)	0.6590 (2)	0.6698 (2)	0.0616 (7)	
H9A	0.7799	0.7281	0.6831	0.074*	
H9B	0.7852	0.6626	0.5926	0.074*	
C10	0.88635 (15)	0.6521 (2)	0.7577 (2)	0.0617 (7)	
H10A	0.9112	0.7044	0.7367	0.074*	
H10B	0.9022	0.6745	0.8331	0.074*	
C11	0.90467 (14)	0.5220 (2)	0.7552 (2)	0.0556 (6)	
H11A	0.9471	0.4953	0.8308	0.067*	
H11B	0.9174	0.5107	0.7030	0.067*	
O1	0.64618 (11)	0.82051 (14)	0.60914 (14)	0.0612 (5)	
O2	0.58007 (14)	0.83408 (18)	0.42662 (16)	0.0852 (7)	
O3	0.59348 (11)	0.98868 (15)	0.52487 (15)	0.0672 (5)	
N1	0.60605 (11)	0.88315 (18)	0.51830 (16)	0.0492 (5)	
O4	0.74570 (10)	0.68205 (14)	0.85048 (13)	0.0533 (4)	
O5	0.81397 (9)	0.66960 (14)	1.03479 (13)	0.0513 (4)	
O6	0.74016 (11)	0.52300 (15)	0.93113 (17)	0.0698 (5)	
N2	0.76639 (11)	0.62317 (16)	0.93912 (16)	0.0447 (5)	

### Atomic displacement parameters (Å^2^)

**Table:**

	*U*^11^	*U*^22^	*U*^33^	*U*^12^	*U*^13^	*U*^23^
N3	0.0448 (10)	0.0328 (9)	0.0462 (10)	0.0007 (8)	0.0243 (9)	−0.0039 (8)
N4	0.0516 (12)	0.0344 (10)	0.0625 (13)	0.0033 (9)	0.0316 (11)	−0.0006 (9)
N5	0.0440 (10)	0.0434 (10)	0.0335 (9)	−0.0035 (8)	0.0193 (8)	−0.0069 (8)
C1	0.0441 (13)	0.0517 (14)	0.0519 (14)	0.0021 (11)	0.0278 (12)	0.0013 (11)
C2	0.0538 (17)	0.0731 (19)	0.0754 (19)	0.0149 (14)	0.0336 (15)	0.0152 (15)
C3	0.0405 (15)	0.110 (3)	0.0671 (19)	0.0017 (17)	0.0243 (14)	0.0035 (18)
C4	0.0516 (17)	0.092 (2)	0.0664 (18)	−0.0206 (16)	0.0319 (15)	−0.0162 (17)
C5	0.0569 (16)	0.0599 (16)	0.0629 (16)	−0.0148 (13)	0.0371 (14)	−0.0149 (13)
C6	0.0439 (13)	0.0464 (13)	0.0459 (13)	−0.0027 (10)	0.0280 (11)	−0.0055 (10)
C7	0.0455 (13)	0.0349 (11)	0.0445 (12)	−0.0014 (9)	0.0270 (11)	−0.0066 (9)
C8	0.0518 (13)	0.0391 (12)	0.0487 (13)	−0.0036 (10)	0.0304 (12)	−0.0098 (10)
C9	0.0662 (17)	0.0392 (13)	0.0772 (18)	−0.0109 (12)	0.0421 (15)	−0.0112 (13)
C10	0.0657 (16)	0.0575 (16)	0.0641 (16)	−0.0226 (13)	0.0404 (14)	−0.0202 (13)
C11	0.0496 (14)	0.0642 (16)	0.0529 (14)	−0.0100 (12)	0.0310 (12)	−0.0077 (12)
O1	0.0807 (12)	0.0462 (10)	0.0502 (10)	0.0166 (9)	0.0361 (10)	0.0115 (8)
O2	0.1258 (19)	0.0759 (14)	0.0539 (12)	−0.0102 (13)	0.0543 (13)	−0.0108 (10)
O3	0.0808 (13)	0.0431 (10)	0.0649 (12)	0.0163 (9)	0.0373 (10)	0.0099 (8)
N1	0.0563 (12)	0.0456 (12)	0.0463 (11)	0.0003 (9)	0.0313 (10)	0.0037 (9)
O4	0.0766 (12)	0.0444 (9)	0.0399 (9)	0.0054 (8)	0.0357 (9)	0.0043 (7)
O5	0.0596 (10)	0.0485 (9)	0.0389 (9)	−0.0026 (8)	0.0261 (8)	0.0033 (7)
O6	0.0716 (12)	0.0441 (10)	0.0778 (13)	−0.0113 (9)	0.0367 (11)	0.0063 (9)
N2	0.0514 (11)	0.0372 (10)	0.0485 (11)	0.0065 (9)	0.0318 (10)	0.0053 (9)

### Geometric parameters (Å, °)

**Table:**

N3—C7	1.331 (3)	C5—H5	0.9300
N3—C6	1.386 (3)	C7—C8	1.499 (3)
N3—H3A	0.8600	C8—C9	1.511 (3)
N4—C7	1.316 (3)	C8—H8	0.9800
N4—C1	1.393 (3)	C9—C10	1.523 (4)
N4—H4A	0.8600	C9—H9A	0.9700
N5—C8	1.499 (3)	C9—H9B	0.9700
N5—C11	1.516 (3)	C10—C11	1.514 (4)
N5—H5A	0.9000	C10—H10A	0.9700
N5—H5B	0.9000	C10—H10B	0.9700
C1—C2	1.382 (4)	C11—H11A	0.9700
C1—C6	1.388 (3)	C11—H11B	0.9700
C2—C3	1.375 (4)	O1—N1	1.274 (2)
C2—H2	0.9300	O2—N1	1.227 (2)
C3—C4	1.384 (4)	O3—N1	1.226 (2)
C3—H3	0.9300	O4—N2	1.270 (2)
C4—C5	1.369 (4)	O5—N2	1.249 (2)
C4—H4	0.9300	O6—N2	1.231 (2)
C5—C6	1.396 (3)		
			
C7—N3—C6	108.93 (18)	N3—C7—C8	127.21 (19)
C7—N3—H3A	125.5	N5—C8—C7	112.84 (17)
C6—N3—H3A	125.5	N5—C8—C9	104.12 (19)
C7—N4—C1	109.12 (19)	C7—C8—C9	115.8 (2)
C7—N4—H4A	125.4	N5—C8—H8	107.9
C1—N4—H4A	125.4	C7—C8—H8	107.9
C8—N5—C11	107.51 (17)	C9—C8—H8	107.9
C8—N5—H5A	110.2	C8—C9—C10	102.4 (2)
C11—N5—H5A	110.2	C8—C9—H9A	111.3
C8—N5—H5B	110.2	C10—C9—H9A	111.3
C11—N5—H5B	110.2	C8—C9—H9B	111.3
H5A—N5—H5B	108.5	C10—C9—H9B	111.3
C2—C1—C6	121.6 (2)	H9A—C9—H9B	109.2
C2—C1—N4	132.3 (2)	C11—C10—C9	104.05 (19)
C6—C1—N4	106.1 (2)	C11—C10—H10A	110.9
C3—C2—C1	116.8 (3)	C9—C10—H10A	110.9
C3—C2—H2	121.6	C11—C10—H10B	110.9
C1—C2—H2	121.6	C9—C10—H10B	110.9
C2—C3—C4	121.5 (3)	H10A—C10—H10B	109.0
C2—C3—H3	119.2	C10—C11—N5	105.2 (2)
C4—C3—H3	119.2	C10—C11—H11A	110.7
C5—C4—C3	122.7 (3)	N5—C11—H11A	110.7
C5—C4—H4	118.7	C10—C11—H11B	110.7
C3—C4—H4	118.7	N5—C11—H11B	110.7
C4—C5—C6	116.0 (3)	H11A—C11—H11B	108.8
C4—C5—H5	122.0	O3—N1—O2	122.4 (2)
C6—C5—H5	122.0	O3—N1—O1	119.45 (19)
N3—C6—C1	106.27 (19)	O2—N1—O1	118.1 (2)
N3—C6—C5	132.2 (2)	O6—N2—O5	120.74 (19)
C1—C6—C5	121.5 (2)	O6—N2—O4	120.96 (19)
N4—C7—N3	109.58 (19)	O5—N2—O4	118.29 (18)
N4—C7—C8	123.10 (19)		
			
C7—N4—C1—C2	−180.0 (3)	C1—N4—C7—N3	−0.9 (3)
C7—N4—C1—C6	0.2 (3)	C1—N4—C7—C8	−177.2 (2)
C6—C1—C2—C3	−0.4 (4)	C6—N3—C7—N4	1.2 (3)
N4—C1—C2—C3	179.8 (3)	C6—N3—C7—C8	177.4 (2)
C1—C2—C3—C4	1.0 (4)	C11—N5—C8—C7	149.12 (19)
C2—C3—C4—C5	−0.9 (5)	C11—N5—C8—C9	22.8 (2)
C3—C4—C5—C6	0.1 (4)	N4—C7—C8—N5	−157.4 (2)
C7—N3—C6—C1	−1.0 (2)	N3—C7—C8—N5	26.9 (3)
C7—N3—C6—C5	−180.0 (2)	N4—C7—C8—C9	−37.6 (3)
C2—C1—C6—N3	−179.4 (2)	N3—C7—C8—C9	146.7 (2)
N4—C1—C6—N3	0.5 (2)	N5—C8—C9—C10	−38.4 (2)
C2—C1—C6—C5	−0.3 (4)	C7—C8—C9—C10	−162.8 (2)
N4—C1—C6—C5	179.6 (2)	C8—C9—C10—C11	39.8 (3)
C4—C5—C6—N3	179.2 (2)	C9—C10—C11—N5	−25.8 (3)
C4—C5—C6—C1	0.4 (4)	C8—N5—C11—C10	2.0 (2)

### Hydrogen-bond geometry (Å, °)

**Table:**

*D*—H···*A*	*D*—H	H···*A*	*D*···*A*	*D*—H···*A*
N3—H3A···O4^i^	0.86	1.93	2.788 (2)	177
N3—H3A···O5^i^	0.86	2.50	3.020 (2)	120
N5—H5B···O1^i^	0.90	1.89	2.771 (2)	167
N5—H5B···O3^i^	0.90	2.64	3.149 (2)	117
N5—H5A···O5^ii^	0.90	1.90	2.768 (2)	162
N4—H4A···O1	0.86	2.04	2.850 (2)	157
N4—H4A···O2	0.86	2.42	3.121 (3)	139

Symmetry codes: (i) −*x*+3/2, *y*−1/2, −*z*+3/2; (ii) *x*, −*y*+1, *z*−1/2.
